# Improving Community Coverage of Oral Cholera Mass Vaccination Campaigns: Lessons Learned in Zanzibar

**DOI:** 10.1371/journal.pone.0041527

**Published:** 2012-07-23

**Authors:** Christian Schaetti, Said M. Ali, Claire-Lise Chaignat, Ahmed M. Khatib, Raymond Hutubessy, Mitchell G. Weiss

**Affiliations:** 1 Department of Epidemiology and Public Health, Swiss Tropical and Public Health Institute, Basel, Switzerland; 2 University of Basel, Basel, Switzerland; 3 Public Health Laboratory Ivo de Carneri, Chake-Chake, Pemba, United Republic of Tanzania; 4 Global Task Force on Cholera Control, World Health Organization, Geneva, Switzerland; 5 Ministry of Health of Zanzibar, Zanzibar, United Republic of Tanzania; 6 Initiative for Vaccine Research, World Health Organization, Geneva, Switzerland; The Australian National University, Australia

## Abstract

**Background:**

Recent research in two cholera-endemic communities of Zanzibar has shown that a majority (∼94%) of the adult population was willing to receive free oral cholera vaccines (OCVs). Since OCV uptake in the 2009 campaign reached only ∼50% in these communities, an evaluation of social and cultural factors and of barriers was conducted to understand this difference for future cholera control planning.

**Methodology/Principal Findings:**

A random sample of 367 adult peri-urban and rural community residents (46.6% immunized vs. 53.4% unimmunized) was studied with a semi-structured interview that inquired about social and cultural features of cholera depicted in a vignette and barriers to OCV uptake. Symptoms (rectal pain, loose skin only in rural community) and perceived causes (uncovered food, contact with contaminated water) specific for severe diarrhea were associated with uptake. Purchasing drugs from pharmacies to stop diarrhea and vomiting was negatively associated with uptake. Increasing household size, age and previous enteric illness episode were positively related to uptake, the latter only at the rural site. The most prominent barrier to uptake was competing obligations or priorities (reported by 74.5%, identified as most important barrier by 49.5%). Next most prominent barriers were lacking information about the campaign (29.6%, 12.2%), sickness (14.3%, 13.3%) and fear of possible vaccine side effects (15.3%, 5.6%). The majority of unvaccinated respondents requested repetition of the vaccination with free OCVs.

**Conclusions/Significance:**

Factors associated with uptake indicated a positive impact of the vaccination campaign and of sensitization activities on vaccine acceptance behavior. Unlike communities opposed to cholera control or settings where public confidence in vaccines is lacking, identified barriers to uptake indicated a good campaign implementation and trust in the health system. Despite prospects and demand for repeating the vaccination, local decision-makers should reconsider how careful logistical arrangements may improve community coverage and thus effectiveness of vaccination campaigns.

## Introduction

Cholera control in populations living at risk of recurrent cholera outbreaks is based on timely treatment and prevention strategies, mainly promoting supply of safe water in sufficient quantities, improved sanitation, and health education (WASH) [1]. Despite these recommendations, cholera has remained a global public health concern; the World Health Organization (WHO) assumes that annual estimates for morbidity and mortality exceed 3 million cases and 100,000 deaths [2]. The WHO also recommends the use of mass oral cholera vaccination as a supplementary prevention measure to WASH [3].

Cholera is an enteric bacterial disease caused by *Vibrio cholerae* serogroup O1 or O139. People living in unsanitary conditions without access to safe drinking water and sanitation are at greatest risk of becoming infected with *V. cholerae*, which is mainly transmitted through the fecal-oral route. Major clinical features, which usually start abruptly after an incubation period of a few hours to five days, include profuse watery diarrhea and vomiting [4]. Without treatment, case-fatality rates may rise to 50% or above. Rehydration is the mainstay for treatment and includes administration of oral rehydration solution (ORS) to patients with mild to moderate symptoms and intravenous fluids to severely dehydrated cases. Antibiotics should also be administered to severe cases to shorten episodes, diminish the amount of intravenous fluids required and reduce shedding of *V. cholerae* [2]. Some studies suggest that antibiotics should also be used for moderate cases [5].

While recent research on the use of oral cholera vaccines (OCVs) in mass vaccination campaigns in endemic communities has focused on epidemiologic parameters [6], [7] and economic aspects [8]–[13], relatively little is known about local perceptions of cholera, intentions to accept OCVs and how such factors are associated with uptake. Even though detailed knowledge of epidemiologic and economic aspects is indispensable for a successful introduction of vaccines, social and cultural factors should also be examined to improve and sustain vaccination coverage [14], [15]. In the past, a lack of attention to community views of illness and prevention has proven to be fatal not only for disease control in certain populations but also for national or international public health goals, e.g., to eradicate polio in Africa [16], [17].

To date, only articles on policymakers' views of cholera [18], [19] and on social factors of oral cholera vaccine uptake [20] have been published, and studies on the feasibility and costs of community mass vaccination campaigns have examined why people might not have taken the OCV [8], [21], [22]. However, an assessment of mass vaccination campaigns to systematically evaluate social and cultural factors associated with OCV uptake and to identify potential barriers is still missing, but likely to be very useful for the benefit of local (and even international) vaccination campaign planning.

Recent research in two cholera-endemic communities of Zanzibar, conducted within the framework of a WHO study to evaluate the use of OCV in endemic settings, has shown that a vast majority (∼94%) of the population targeted for the campaign was in principle willing to receive free vaccines against cholera [23]. Since actual OCV acceptance (or uptake) reached only ∼50% in this pre-vaccination sample, an evaluation of social and cultural factors and of barriers to OCV uptake was needed to understand this difference for future cholera control planning in Zanzibar.

Findings reported here are based on the research approach of cultural epidemiology, which was used in pre-vaccination studies to examine social and cultural determinants of anticipated and actual OCV acceptance [23], [24]. Cultural epidemiology [25] is a research approach in health social sciences based on Arthur Kleinman's framework of illness explanatory models [26]. The approach integrates quantitative and qualitative data to study community views of illness [27]–[31] and how these influence health-related behavior [32]–[34]. It has been validated in numerous settings to study mental ill-health, chronic and infectious diseases and health-related stigma and involves use of culturally adapted semi-structured interviews to elicit the distribution of locally valid features of illness-related experience, meaning and behavior [35].

This study examined data from a peri-urban and a rural community targeted in the 2009 OCV mass vaccination campaign in Zanzibar. It aimed to evaluate the influence of social and cultural factors on OCV uptake and to identify logistical, medical, social and system-related barriers to uptake, stratified by site and by gender.

## Methods

### Ethics statement

The Research Ethics Review Committee of the World Health Organization and the Ethics Committee of the Ministry of Health of Zanzibar approved this study. Participants were informed orally about this study and also given a detailed information sheet. Only those who gave written consent were interviewed. No compensation was offered for the interview. Interview data sheets did not bear the names of respondents and all data were anonymized before analysis.

### Setting

The east African archipelago of Zanzibar belongs to the United Republic of Tanzania and is inhabited by ∼1.2 million people who are predominantly Muslim. Kiswahili is the main language, but English is also widely used. The archipelago is located ∼60 km off the coast of mainland Tanzania and consists of two major islands—Unguja in the south and Pemba in the north—and several islets; it can be reached from the coast by ferry or air within 20 minutes to 2 hours. Zanzibar has been regularly affected by cholera; the first cases in recent times were detected in 1978 [36], [37].

This study was conducted in the peri-urban Shehia of Chumbuni (population ∼11,000) in Unguja and the rural Shehia of Mwambe (∼8,000) in Pemba. Both Shehias (administrative term for community in Zanzibar) were among the core areas of a mass vaccination campaign that was conducted in early 2009 by the Ministry of Health of Zanzibar (MoH) with support from the WHO. The sample for this study was drawn from these two Shehias because they had been studied in a pre-vaccination survey in 2008 [23], [24]. The peri-urban site, an unplanned, slum-like extension of the capital, mainly consists of brick houses and is characterized by a high population density; the rural site consists of hamlets and most people live in mud houses. More details of both sites have been reported elsewhere [24].

### Mass vaccination campaign

The mass vaccination campaign aimed to vaccinate ∼50,000 inhabitants with Dukoral®, a two-dose OCV containing killed *V. cholerae* O1 bacteria and recombinant cholera toxin B subunit protein [38]. Dukoral® was the only OCV pre-qualified by the WHO at the time of vaccination. It requires a cold chain for storage and safe water (∼1.5 dl per dose) for its administration. It was offered without charge in two rounds from January 17 to 26 and February 7 to 16, 2009, to residents aged two years or older from six Shehias from Unguja and Pemba that had been identified as recent cholera hotspots. Nine vaccination posts were set up on each island that operated daily for at least eight hours and were staffed with local healthcare workers and villagers.

Information activities for the campaign started with a meeting with district officials on December 23, 2008, followed by three meetings to inform leaders, Shehia committee members and mobilizers from each community (January 5 and 10, 2009) and general community residents (January 15, 2009) (MoH, Health Promotion Unit, OCV Social Mobilization Report, February 20, 2009). A refresher meeting in the communities followed shortly before the second round on February 5, 2009. Social mobilization used posters, leaflets, street banners and T-shirts to disseminate information on the OCV campaign and to reinforce general hygiene and sanitation messages in the six Shehias. Messages were continuously broadcast on national TV and radio from the first until the last day of the campaign. The local press was also briefed and newspaper articles reported from the campaign to promote participation. The campaign was officially launched by the Minister of Health who drank the vaccine publicly at the Chumbuni Primary Healthcare Unit (*Zanzibar Today*, January 18, 2009). Mobilizer teams were formed for each Shehia and delivered information from house to house and by megaphone. Each team consisted of five to six community residents representing also women's groups, youth, religious groups and members of the opposition party. Key messages highlighted not only the importance of vaccination for cholera prevention, but also promoted hygiene messages to prevent other diarrheal diseases, and explained administration of the OCV, its characteristic features and potential for mild side effects.

### Design and sampling

This was a cross-sectional interview survey based on a case-control design to identify factors associated with vaccine uptake among vaccinated and unvaccinated community members targeted for the mass vaccination campaign. In addition, unvaccinated participants were also interviewed about barriers to uptake for site- and gender-related comparative analysis. Data were collected in June and July 2009, six months after the mass vaccination campaign. The sampling frame for this study was derived from the census database that had been compiled by the International Vaccine Institute shortly before the mass vaccination campaign implementation in early 2009 [39]. Names, age, sex, OCV vaccination status and a unique house identification number were extracted for both study Shehias. Respondents' houses in Chumbuni were located with the help of aerial photographs indicating house numbers; houses in Mwambe were located with the help of local assistants.

Approximately 380 adults, based on a sample size of 330 [40] with 15% compensation for missing data, were identified following a stratified random sampling procedure. After exclusion of respondents who had been interviewed before the vaccination for the baseline study [24], all respondents aged 18 years and older were selected. Second, peri-urban and rural respondents were separated and groups of women and men created among them. Third, of the approximately 95 women and 95 men required per site, 50% were selected from those who had received two doses of the OCV, 40% from those who had not received a single dose and 10% from those with one dose only. Only residents who were physically and mentally fit to stand an interview of approximately one hour duration were included in the sample. Women who had not taken the vaccine because of pregnancy during the mass vaccination campaign were not interviewed.

### Instrument

Semi-structured interviews based on the Explanatory Model Interview Catalogue (EMIC) are the principal instrument for cultural epidemiological studies and elicit locally valid features of illness-related experience (operationalized as categories of distress), meaning (perceived causes) and behavior (help seeking) [25], [35]. An EMIC interview for study of diarrhea-free community residents was developed based on the pre-vaccination survey [24] (see supporting information, Text S1). A ten-day workshop was conducted shortly before the survey to train field workers and pilot the EMIC interview in Shehias adjacent to the study communities.

After recording relevant socio-demographic characteristics, interviews began with the telling of a brief story in easily understandable terms, making use of a clinical vignette that described a cholera patient with cardinal somatic symptoms. To study socio-cultural features of cholera-like illness, respondents were asked a series of open and closed questions. These elicited patterns of distress (i.e., respondents' opinions on what additional physical symptoms the cholera patient described in the vignette might suffer from, and how the illness might impact him/her socially, emotionally and financially), perceived causes (i.e., what causes the illness may be attributed to) and help-seeking behavior (i.e., what would usually be done at the patient's home for self treatment and what sources of help would be consulted outside the household).

Respondents who did not swallow two doses of the OCV during the mass vaccination campaign were queried about their reasons against vaccination by specifically inquiring about barriers related to logistical, social and system-relevant and medical aspects.

### Data management and analysis

#### Data entry

Quantitative data were recorded by interviewers on data sheets, double entered in Epi Info 3.5.1 (CDC, Atlanta, GA, USA) by data entry clerks and cleaned for statistical analysis in SAS 9.2 (SAS Institute, Cary, NC, USA). Qualitative data were written down during the interview by note takers in Kiswahili (or in English in a few cases). After translation into English, narratives were typed in a pre-coded word processor template that reflected interview items; this procedure followed the pre-vaccination survey [23]. This enabled automatic importation of entire interviews with codes into the qualitative data analysis software MAXQDA 10 (VERBI Software, Consult. Sozialforschung. GmbH, Marburg, Germany). For integrated analysis of quantitative and qualitative data, quantitative variables (see below) were imported into MAXQDA 10; this made it possible to retrieve narrative segments based on analytically relevant findings or statistical relationships.

#### Multivariable analysis of factors of uptake

Socio-demographic characteristics were coded as numeric or categorical variables. Categories of socio-cultural features of cholera-like illness were assigned a value of 2 if they were mentioned spontaneously and a value of 1 if they were mentioned only after probing. Those among the reported categories that were identified as single most troubling (among patterns of distress), most important (perceived causes) or most helpful (help seeking) were given an additional value of 3. A cumulative prominence was then calculated for each category ranging from 0 to 5. This approach based on the ranked prominence of responses has been widely used in analytic cultural epidemiological studies, which have examined how socio-cultural features of illness affect health behavior [32], [34].

To identify social and cultural factors explaining OCV uptake, a multivariable logistic regression model was calculated. The outcome variable (i.e., OCV vaccination status) was obtained from mass vaccination campaign data that had been compiled electronically during the campaign [39]. Based on the recommended schedule for Dukoral® requiring two doses for full protection, respondents who had received two doses were coded as 1 (“vaccinated”) and those who had received only one or no dose were coded as 0 (“unvaccinated”). The regression analysis included interaction with site as suggested by site-specific findings from the pre-vaccination survey [24] and because OCV uptake was higher in the rural than in the peri-urban site (58.8% vs. 40.8%, p = 0.001).

Only explanatory variables reported by 5–95% were considered for analysis. Following the approach taken in the pre-vaccination survey [23], variables whose univariable association with OCV uptake had a p<0.2 were identified first. Second, multivariable regression models related to patterns of distress, perceived causes and help seeking were run by considering only variables that were retained in the first step. Each of these sub-models was adjusted for socio-demographic characteristics. To calculate the final model, only those variables which were retained with a p<0.2 in these sub-models were considered. Interaction between each explanatory variable and site (rural vs. peri-urban site at baseline) was tested in sub-models; only interaction terms retained with a p<0.1 in sub-models were used in the final model. The final model reports adjusted odds ratios with 95% confidence intervals and p values. In case of significant interaction with site, site-specific estimates are presented.

#### Descriptive analysis of barriers to uptake

Coding and calculation of variables related to barriers followed the approach used for socio-cultural features of illness. Unvaccinated respondents' spontaneous and probed answers for each barrier and the barrier they identified as most important were recorded. Thematically similar barriers were subsumed under groups of logistical, medical and social/system-related barriers. The non-parametric Wilcoxon test was used for identifying statistically significant differences of prominence between both sites and between genders.

## Results

### Sample characteristics

A total of 378 respondents were interviewed. Eleven interviews were excluded from analysis due to pregnancy. Of the remaining 367 respondents, 46.6% were vaccinated with two doses, 9.3% with one dose only and 44.1% had not drunk any dose of OCV. Their characteristics are presented in Table 1. All respondents were Muslims and of Tanzanian nationality.

**Table 1:** Socio-demographic characteristics and vaccination status of a sample interviewed after a community mass vaccination campaign in Zanzibar, stratified by site and gender.

	Total	Peri-urban site	Rural site		Women	Men	
Number (%)	367 (100)	189 (51.5)	178 (48.5)		180 (49.0)	187 (51.0)	
***Age (years)***							
Mean (SD)	35.4 (14.6)	33.1 (13.5)	37.8 (15.4)	**	35.7 (13.8)	35.1 (15.4)	
Median (range)	32 (18–90)	28 (18–75)	36.5 (18–90)	**	33 (18–90)	30 (18–80)	
***Marital status (%)***							
Never married	30.5	42.9	17.4	***	21.1	39.6	***
Married	59.4	49.7	69.7	***	61.1	57.8	
Separated	0.5	0.0	1.1		0.0	1.1	
Divorced	6.5	6.9	6.2		12.2	1.1	***
Widowed	3.0	0.5	5.6	**	5.6	0.5	**
***Household size (number of persons)***							
Mean (SD)	6.9 (3.0)	7.6 (3.2)	6.3 (2.6)	***	6.8 (3.0)	7.1 (2.9)	
Median (range)	7 (1–15)	7 (1–15)	6 (1–13)	***	7 (1–14)	7 (1–15)	
***Main occupation (%)***							
Agriculture	30.8	3.2	60.1	***	35.0	26.7	
Fishing	6.0	0.0	12.4	***	0.0	11.8	***
Self-employment	23.7	36.0	10.7	***	18.3	28.9	*
Formal employment	8.2	13.2	2.8	***	3.3	12.8	***
Housewife	12.3	18.0	6.2	***	25.0	0.0	***
Casual laborer	0.8	1.1	0.6		0.0	1.6	
Student	12.5	18.0	6.7	**	12.2	12.8	
Not active/retired	5.7	10.6	0.6	***	6.1	5.3	
***Highest education (%)***							
No education	8.4	4.8	12.4	*	10.6	6.4	
Koranic school	23.7	10.1	38.2	***	30.6	17.1	**
Primary school	26.4	21.2	32.0	*	19.4	33.2	**
Secondary school	36.8	56.1	16.3	***	35.6	38.0	
Above secondary school	4.6	7.9	1.1	**	3.9	5.3	
Vocational school	1.4	1.6	1.1		0.6	2.1	
Higher education	3.3	6.3	0.0	***	3.3	3.2	
***Household income (%)***							
More regular and dependable	39.8	54.0	24.7	***	41.1	38.5	
Less regular and dependable	60.2	46.0	75.3		58.9	61.5	
***Vaccination status***							
Receipt of 2 doses, number (%)	171 (46.6)	86 (45.5)	85 (47.8)		85 (47.2)	86 (46.0)	
Receipt of 1 dose, number (%)	34 (9.3)	18 (9.5)	16 (9.0)		17 (9.4)	17 (9.1)	
Receipt of 0 doses, number (%)	162 (44.1)	85 (45.0)	77 (43.3)		78 (43.3)	84 (44.9)	

SD: Standard deviation, t test used for comparing means, Wilcoxon test used for comparing medians, Fisher's exact test used for comparing proportions (*p<0.05, **p<0.01, ***p<0.001).

### Social and cultural factors associated with OCV uptake

Multivariable logistic regression analysis identified socio-cultural features of cholera-like illness associated with OCV uptake, adjusted for socio-demographic characteristics (Table 2). Among categories of distress, two of the somatic symptoms that were mentioned in connection with the cholera vignette were positively associated with OCV uptake: rectal pain and loose or shriveled skin, which is a sign of dehydration. Rectal pain was spontaneously reported by 1.9% and mentioned by 68.7% upon probing. Vaccinated and unvaccinated respondents explained that this symptom meant that frequent passing of stool may be painful to the person described in the vignette. Loose or shriveled skin was only associated with vaccine uptake among rural respondents. It was reported by 86.6% of the total sample; 88.8% reported it in the rural and 84.7% in the peri-urban site and more rural respondents mentioned it spontaneously (33.1%) compared to peri-urban respondents (5.8%). Accounts from vaccinated and unvaccinated respondents were similar, saying that frequent diarrhea leads to loss of water in the body, which in turn was seen as the reason for dehydration manifested by the sign of loose skin.

**Table 2:** Social and cultural factors associated with oral cholera vaccine uptake in a community mass vaccination campaign in Zanzibar, n = 367.

	Adjusted analysis^a^				
	OR^b^	95% CI^c^		p value^d^	Int^e^
***Categories of distress: somatic symptoms***					
Pus in stool	1.35	0.87	2.11	0.178	
Rectal pain	1.83	1.17	2.86	**0.008**	
Sunken eyes	1.20	0.86	1.67	0.289	
Loose skin (peri-urban site)	0.62	0.32	1.20	0.157	
Loose skin (rural site)	2.00	1.18	3.40	**0.010**	**
***Perceived causes***					
Unprotected/spoiled food	1.24	1.00	1.54	**0.049**	
Contact with contaminated water	1.43	1.06	1.92	**0.019**	
***Outside help seeking***					
Pharmacy/Over-the-counter drugs	0.55	0.34	0.88	**0.013**	
***Socio-demographics and previous illness episode***					
Age	1.03	1.01	1.04	**0.002**	
Household size	1.09	1.01	1.18	**0.028**	
Site (rural vs. peri-urban)	0.28	0.10	0.77	**0.014**	
Previous enteric illness episode (peri-urban site)	0.69	0.18	2.65	0.586	
Previous enteric illness episode (rural site)	3.03	1.13	8.09	**0.027**	*

a List of variables with univariable association with vaccine uptake at p<0.2 that were included in adjusted models. Gender, a matching variable, was not included as a main factor because its p value was above 0.2; site was included for interaction testing (see footnote e);

b Adjusted odds ratio;

c 95% Confidence interval;

d Figures in bold if p<0.05;

e Interaction of site (rural with peri-urban baseline) was considered if the p value of the interaction term was less than 0.1 (*p<0.1, **p<0.01).

Among categories of perceived causes, two categories were positively associated with OCV uptake: eating food that has not been covered properly and contact with contaminated water. The first category was mentioned by 89.4% of the total sample and identified by 8.2% as most important cause for cholera. Among those who reported this category, the majority said that if food is not covered properly, flies or other insects that carry germs may contaminate it. A 22-year-old farmer from Pemba, who had ingested both doses, explained it this way: *“Yes, this is the area where one can get it [the illness described in the vignette], because the flies are carrying feces and land with it on the food.”* Such explanations were not only typical for the vaccinated group because narratives from unvaccinated respondents also frequently showed flies as main disease vector.

Fewer respondents (69.2%) reported that contact with contaminated water was a cause for cholera, and only 1.9% identified it as most important cause. Both vaccinated and unvaccinated respondents referred to dirty water as a potential cause because it contains bacteria or other disease-causing organisms that can be transmitted through the fecal-oral route. The following example from a 19-year-old fully immunized male student from Unguja illustrates this reasoning: *“Yes, because it is already contaminated with bacteria. If you have touched the water and not washed your hands with soap and then you eat food you will get the disease.”*

Among categories of help seeking outside the home, consulting pharmacies was negatively associated with OCV uptake. While everybody reported spontaneously that a patient with cholera-like illness should be sent to professional health facilities, 32.4% of the sample also reported getting drugs from the pharmacy as a means to stop diarrhea and vomiting, though none of them identified this category as most helpful. Primarily antibiotics like tetracycline or septrine were mentioned among both vaccinated and unvaccinated groups.

Among socio-demographic characteristics, increasing household size and increasing age was positively related to OCV uptake. A total of 9.5% reported a household episode of the illness described in the cholera vignette; rural respondents reported more such episodes than their peri-urban counterparts (13.5% vs. 5.8%, p = 0.013). This variable was also included in the analysis and showed a positive association with OCV uptake at the rural site.

### Barriers to OCV uptake

All 196 respondents who were not completely immunized were asked the following open question: “Can you tell us the reasons why you did not swallow two doses of the cholera vaccine?” Individual and grouped barriers are presented for the overall sub-sample of unvaccinated respondents (Table 3), and stratified by site (Table 4) and by gender (Table 5).

**Table 3:** Barriers to uptake of an oral cholera vaccine in a community mass vaccination campaign in Zanzibar.

	Pooled sample, n = 196			
	How reported?			
Barriers to uptake^a^	Total^b^ %	Spontaneous^c^ %	Most important^d^ %	Mean prom.^e^
***Logistical barriers***	***76.5***	***71.4***	***63.3***	***3.38***
Competing obligations/priorities	74.5	69.4	49.5	2.92
Lacking information about campaign	29.6	9.7	12.2	0.76
Vaccination post open days/hours limited	13.8	3.1	1.5	0.21
Costs apart from vaccine	3.6	0.5	0.0	0.04
Organizational problems at vaccination post	0.5	0.0	0.0	0.01
***Medical barriers***	***31.1***	***23.5***	***23.5***	***1.25***
I was sick (not due to vaccine)	14.3	14.3	13.3	0.68
Fear of possible side effects from vaccine	15.3	6.1	5.6	0.38
Doubted effectiveness of vaccine	9.2	5.6	4.6	0.29
***Social/system-related barriers***	***12.2***	***4.1***	***3.6***	***0.27***
Vaccine free of charge (useless medicine)	5.6	2.6	1.0	0.11
Fear of infertility	3.1	0.0	1.5	0.08
Mistrust motives of campaign	5.1	2.0	0.0	0.07
Social pressure against taking vaccine	3.1	1.0	0.5	0.06
Lacking confidence in government	2.6	1.0	0.5	0.05
Prior bad experience with health system	0.5	0.0	0.0	0.01
***Miscellaneous***	***8.7***	***8.7***	***6.6***	***0.37***
Other barriers	7.1	7.1	5.6	0.31
Cannot say/Nothing	1.5	1.5	1.0	0.06

a Barriers ordered according to descending mean prominence (see footnote e), grouped barriers in bold;

b Percentage of barriers reported spontaneously and after probing;

c Percentage of barriers reported spontaneously only;

d Percentage of barriers that were identified as single most important among all the reported barriers. Six respondents who only received one dose identified barriers that were not among the ones listed as most important: four respondents reported “Experience of side effects from first dose of vaccine,” two respondents reported “Did not have information about timing of second dose;”

e Mean prominence based on values assigned for each barrier (3 = identified as most important, 2 = reported spontaneously, 1 = reported after probing, 0 = not reported).

**Table 4:** Barriers to uptake of an oral cholera vaccine in a community mass vaccination campaign in Zanzibar, stratified by site.

	Peri-urban site, n = 103				Rural site, n = 93			
	How reported?				How reported?			
Barriers to uptake^a^	Total^b^ %	Spontaneous^c^ %	Most important^d^ %	Mean prom.^e^	Total^b^ %	Spontaneous^c^ %	Most important^d^ %	Mean prom.^e^
***Logistical barriers***	***79.6***	***75.7***	***68.0***	***3.59***	***73.1***	***66.7***	***58.1***	***3.14***
Competing obligations/priorities	76.7	73.8	55.3	3.17	72.0	64.5	43.0	2.66
Lacking information about campaign	20.4	8.7	11.7	0.64	39.8	10.8	12.9	0.89
Vaccination post open days/hours limited	8.7	4.9	1.0	0.17	19.4	1.1	2.2	0.27
Costs apart from vaccine	2.9	0.0	0.0	0.03	4.3	1.1	0.0	0.05
Organizational problems at vaccination post	0.0	0.0	0.0	0.00	1.1	0.0	0.0	0.01
***Medical barriers***	***27.2***	***22.3***	***23.3***	***1.19***	***35.5***	***24.7***	***23.7***	***1.31***
I was sick (not due to vaccine)	15.5	15.5	14.6	0.75	12.9	12.9	11.8	0.61
Fear of possible side effects from vaccine	10.7	4.9	5.8	0.33	20.4	7.5	5.4	0.44
Doubted effectiveness of vaccine	5.8	3.9	2.9	0.18	12.9	7.5	6.5	0.40
***Social/system-related barriers***	***8.7***	***2.9***	***1.0***	***0.15***	***16.1***	***5.4***	***6.5***	***0.41***
Vaccine free of charge (useless medicine)	1.9	1.0	0.0	0.03	9.7	4.3	2.2	0.20
Fear of infertility	1.9	0.0	0.0	0.02	4.3	0.0	3.2	0.14
Mistrust motives of campaign	1.9	1.0	0.0	0.03	8.6	3.2	0.0	0.12
Social pressure against taking vaccine	3.9	1.9	1.0	0.09	2.2	0.0	0.0	0.02
Lacking confidence in government	0.0	0.0	0.0	0.00	5.4	2.2	1.1	0.11
Prior bad experience with health system	0.0	0.0	0.0	0.00	1.1	0.0	0.0	0.01
***Miscellaneous***	***6.8***	***6.8***	***5.8***	***0.31***	***10.8***	***10.8***	***7.5***	***0.44***
Other barriers	5.8	5.8	4.9	0.26	8.6	8.6	6.5	0.37
Cannot say/Nothing	1.0	1.0	1.0	0.05	2.2	2.2	1.1	0.08

a Barriers ordered according to descending mean prominence for the pooled sample (see Table 3), grouped barriers in bold;

b Percentage of barriers reported spontaneously and after probing;

c Percentage of barriers reported spontaneously only;

d Percentage of barriers that were identified as single most important among all the reported barriers;

e Mean prominence based on values assigned for each barrier (3 = identified as most important, 2 = reported spontaneously, 1 = reported after probing, 0 = not reported), *p<0.05 (Wilcoxon test for comparison of mean prominence between site).

**Table 5:** Barriers to uptake of an oral cholera vaccine in a community mass vaccination campaign in Zanzibar, stratified by gender.

	Women, n = 95				Men, n = 101				
	How reported?				How reported?				
Barriers to uptake^a^	Total^b^ %	Spontaneous^c^ %	Most important^d^ %	Mean prom.^e^	Total^b^ %	Spontaneous^c^ %	Most important^d^ %	Mean prom.^e^	
***Logistical barrier*** *s*	***66.3***	***62.1***	***56.8***	***2.99***	***86.1***	***80.2***	***69.3***	***3.74***	*
Competing obligations/priorities	65.3	61.1	47.4	2.68	83.2	77.2	51.5	3.15	
Lacking information about campaign	26.3	7.4	8.4	0.59	32.7	11.9	15.8	0.92	
Vaccination post open days/hours limited	8.4	1.1	1.1	0.13	18.8	5.0	2.0	0.30	*
Costs apart from vaccine	2.1	0.0	0.0	0.02	5.0	1.0	0.0	0.06	
Organizational problems at vaccination post	0.0	0.0	0.0	0.00	1.0	0.0	0.0	0.01	
***Medical barriers***	***38.9***	***32.6***	***32.6***	***1.69***	***23.8***	***14.9***	***14.9***	***0.83***	**
I was sick (not due to vaccine)	26.3	26.3	24.2	1.25	3.0	3.0	3.0	0.15	***
Fear of possible side effects from vaccine	15.8	6.3	5.3	0.38	14.9	5.9	5.9	0.39	
Doubted effectiveness of vaccine	5.3	3.2	3.2	0.18	12.9	7.9	5.9	0.39	
***Social/system-related barriers***	***7.4***	***0.0***	***1.1***	***0.11***	***16.8***	***7.9***	***5.9***	***0.43***	*
Vaccine free of charge (useless medicine)	1.1	0.0	0.0	0.01	9.9	5.0	2.0	0.21	**
Fear of infertility	2.1	0.0	1.1	0.05	4.0	0.0	2.0	0.10	
Mistrust motives of campaign	2.1	0.0	0.0	0.02	7.9	4.0	0.0	0.12	
Social pressure against taking vaccine	3.2	0.0	0.0	0.03	3.0	2.0	1.0	0.08	
Lacking confidence in government	0.0	0.0	0.0	0.00	5.0	2.0	1.0	0.10	*
Prior bad experience with health system	0.0	0.0	0.0	0.00	1.0	0.0	0.0	0.01	
***Miscellaneous***	***9.5***	***9.5***	***7.4***	***0.41***	***7.9***	***7.9***	***5.9***	***0.34***	
Other barriers	8.4	8.4	6.3	0.36	5.9	5.9	5.0	0.27	
Cannot say/Nothing	1.1	1.1	1.1	0.05	2.0	2.0	1.0	0.07	

a Barriers ordered according to descending mean prominence for the pooled sample (see Table 3), grouped barriers in bold;

b Percentage of barriers reported spontaneously and after probing;

c Percentage of barriers reported spontaneously only;

d Percentage of barriers that were identified as single most important among all the reported barriers;

e Mean prominence based on values assigned for each barrier (3 = identified as most important, 2 = reported spontaneously, 1 = reported after probing, 0 = not reported), *p<0.05, **p<0.01, ***p<0.001 (Wilcoxon test for comparison of mean prominence between gender).

#### Most prominent barriers

Logistical factors were reported as paramount barriers, followed by medical issues; social and system-related factors were the least prominent barriers (Table 3). The most prominent individual barrier to OCV uptake (i.e., the one having the highest mean prominence) was *competing obligations or priorities*, which was reported by almost three-quarters (74.5%) of the unvaccinated respondents and identified by nearly half (49.5%) as the most important barrier. Analysis of qualitative data from these respondents indicated that they had mostly been away for a longer time on the mainland or another island and thus were less able to reach the vaccination posts. Activities included working in farms, going on month-long fishing trips and some visited their relatives or were away from home for study or exams.

The second most prominent barrier was *lacking information about the campaign*, reported by 29.6% and identified as most important barrier by 12.2%. Almost everyone who did not have information about the campaign also reported his/her absence because of other activities. The following accounts illustrate how lacking information and being away together prevented vaccine uptake. Respondents were either away during both rounds, as illustrated by the account of a 36-year-old man from Chumbuni: *“I was not here during the campaign and I didn't know when the campaign started and finished. I am a seaman. My wife informed me that all the people in the house got the vaccine. The day I arrived here I was advised to take the vaccine but I didn't take it because I was tight with other activities. And on the second day my boss asked me to go to Mombasa.”* Or they were only in their village during the second round, but not given the vaccine: *“I was not around because I had traveled to Wete. And when I came back I went to the vaccination post and the workers told me that I cannot get it because I missed the first dose.”* (Housewife from Mwambe, 50 years old)

*Sickness*, which was reported by 14.3% of unvaccinated respondents in total and identified by 13.3% as the single most important reason, was the third most prominent barrier to uptake. Respondents who reported a sickness were either uncomfortable to take the vaccine, concerned about a potential negative impact of vaccination on their health, or simply not able to access vaccination posts because of a physical handicap or a recent delivery or surgery.

*Fear of possible side effects* was the fourth most prominent barrier against vaccination, reported by 15.3% in total and identified by 5.6% as most important. People were afraid of side effects such as diarrhea, vomiting, nausea, skin reactions after injection, and exacerbations of underlying diseases due to interaction with the vaccine. Also, something free of charge was believed to cause problems. Three respondents were afraid of side effects if the vaccine was administered concurrently with other drugs—they were also among those who reported being sick as main barrier to vaccination.

Besides fear of side effects, *doubted effectiveness of the vaccine* was reported by 9.2% as another vaccine-related barrier; and for 4.6% this category was the main reason against taking the OCV. Respondents were not sure about the benefit for their own health or the effectiveness of the vaccine.

#### Least prominent barriers

The four least prominent barriers to OCV uptake were related to *lacking confidence in the government* (reported in total by 2.6% of the unvaccinated sub-sample), *costs apart from the vaccine* (3.6%), *prior bad experience with health system* (0.5%), and *organizational problems at vaccination post* (0.5%) (Table 3).

Other barriers related to social issues were reported by 5% or less: *mistrust motives of the campaign* were reported by 5.1% and *social pressure against vaccination* and *fear of infertility* by 3.1%. Nobody reported that discouragement by authoritative persons made them refuse vaccination.

#### Site- and gender-specific barriers

Among the most prominent barriers, *lacking information about the campaign* was more often reported and identified as most important barrier among rural than peri-urban respondents (p = 0.012) (Table 4). Narratives indicated that rural respondents were away from their homes for longer times and several went fishing for months, which made it difficult for them to be home at the right time slot needed for the vaccination: *“I had traveled to Unguja for fishing for a period of one month and fifteen days. And I had no information about the vaccine campaign.”* (Unvaccinated rural fisherman, 35 years old)

Four more barriers were more prominent in the rural area: *limited open days/hours of vaccination post* (p = 0.045), *vaccine free of charge is useless* (p = 0.020), *mistrust motives of the campaign* (p = 0.037) and *lacking confidence in government* (p = 0.019). Three out of these five rural barriers were also reported with more prominence by men (in the total sub-sample): Limited open days/hours of vaccination post (p = 0.032), *vaccine free of charge is useless* (p = 0.008) and *lacking confidence in government* (p = 0.030).

The analysis of grouped categories showed that men reported significantly more logistical, social and system-related barriers than women (Table 5). Narratives indicated that men had their business or were committed to fishing and farming and mostly away during the daytime or for months. These commitments limited access to vaccination posts because open hours were too limited or because the duration of the campaign itself was not long enough. Even though women reported fewer such problems, they also explained their absence as being too busy with work and thus unable to reach the posts in time. Mostly rural men, compared to only one woman from the peri-urban site, complained about why the vaccine was offered free of charge despite the fact that other drugs require purchase. This and the finding that only rural men were not confident about the government's intentions is illustrated by the account of a 40-year-old man from Pemba: *“I did not drink the medicine because I felt it does not help and drugs are not given free. Also when they give it to you free of charge there is some reason for doing that.”*

*Sickness* was equally prominent in both sites, but the majority who reported this barrier were women (26.3% vs. 3.0%, p<0.001). Qualitative data showed that many of those women had actually been eager to receive the vaccine, but could not because of troubling symptoms or because they were afraid that the vaccine could make their present condition worse. A 30-year-old housewife from Chumbuni explained why she could not take the vaccine because of her severe fever: *“I came home during the vaccination days but I had severe fever and I left soon after the campaign. While I was there I heard an announcement about the vaccination on the radio. But because of my condition—I was still sick—I was unable to come and take the vaccine.”*

Three men only reported sickness, but identified it as the most important barrier; they exclusively referred to a perceived harmful interaction between drugs and the OCV: *“I was sick with severe fever. And they told me it was [high blood] pressure. I was using many drugs and therefore I was told not to mix drugs because of harmful effects.”* (50-year-old farmer in Mwambe)

Since the majority of respondents (76.5%) had missed the vaccination due to logistical constraints (Table 3), further analysis of their views was deemed necessary. At the end of the interview, respondents were encouraged to share any additional comments, advice or suggestions about the health problems and vaccines that had been discussed or needed to be emphasized. Based on the assumption that these respondents did not object to receiving the OCV in principle, thematic analysis of their concluding statements was done.

Most of the respondents who missed the complete course of vaccination because of logistical barriers requested the government repeat the vaccination to make them fully immunized and to vaccinate those people who did not get the vaccine during the campaign. They also emphasized the need to make the vaccine available free of charge and frequently demanded more health education in the communities. Even though men reported logistical barriers more prominently (Table 5), themes identified in male and female narratives were very similar. A businessman from Chumbuni, aged 28 years, gave the following advice: *“I would like to advise the Ministry of Health to provide free vaccines. They should also sensitize the community by providing health education. This will make the community aware of the importance of vaccines.”* A female student from Mwambe, aged 18 years, also suggested how to improve the campaign: *“The vaccination should be repeated so that I can also make it. But I suggest that we should be better informed about the real date of the second dose.”*

Because campaign implementers had paid attention to minimize accessibility-related barriers, issues of distance to vaccination posts were not specifically elicited as potential barrier. Thematic analysis of accounts of unvaccinated respondents did not reveal that difficulties with travel to reach the vaccination posts may have been a problem, thereby corroborating this assumption.

## Discussion

This post-vaccination survey clarified social and cultural factors of uptake of an oral cholera vaccine in a peri-urban and a rural community of Zanzibar. Socio-cultural features of cholera-like illness and socio-demographic factors were identified, and logistical, medical and social and system-related barriers were examined among unvaccinated community residents.

### Influence of social and cultural factors on uptake

Compared to the pre-vaccination analysis of determinants of OCV uptake where nonspecific symptoms of cholera determined uptake negatively, rectal pain was positively associated with OCV uptake in this survey. Even though cholera-related purging is usually painless [4], this finding may indicate a priority for vaccines not only for cholera but also for severe diarrhea in general. Features of dehydration were identified as promoting factors for vaccination in both pre- and post-vaccination surveys. However, while unconsciousness determined uptake positively in the pre-vaccination study in both sites, reporting a loose or shriveled skin influenced only rural respondents to take the OCV.

Recognizing biomedical risk factors for cholera (i.e., the potential risk for infection with germs when leaving food uncovered or when coming into contact with contaminated water) prompted respondents to take the OCV. This may reflect the positive impact of the mass vaccination campaign on people's ideas and behavior. Neither biomedical nor alternative factors that had been perceived to cause cholera were identified as determinants of OCV uptake in the pre-vaccination survey.

Even though the OCV was offered for free during the campaign and no considerable direct costs were likely to be incurred in accessing the vaccination posts, purchasing drugs in pharmacies to stop vomiting and diarrhea competed with vaccines. This finding may indicate that the idea of treating cholera with drugs seemed to be more attractive than prevention with vaccination, or that the appeal of well-known powerful antibiotics was so valued that they overrode vaccination as a new and more uncertain intervention for cholera in Zanzibar.

Reporting a previous enteric illness episode at the rural site was positively associated with uptake. This confirms results from a study in Vietnam [11], but contrasts the pre-vaccination study in Zanzibar, where reporting of such episodes did not determine vaccine uptake. This finding nevertheless suggests a higher perceived need for vaccination in the rural area, which is supported by the higher OCV uptake among rural respondents and the finding from the pre-vaccination study that fear of disruptions of healthcare services during cholera outbreaks was a positive determinant of OCV uptake in the rural area. Consistent with the pre-vaccination study is the finding that older people were more likely to drink the OCV. A higher household size, which had made people less willing to pay for an OCV before the campaign, was positively associated with uptake; this might demonstrate the higher perceived need for vaccines if no costs are attached to it.

### Assessment of barriers to uptake

Logistical issues were paramount barriers against taking the vaccine. Issues around social pressure or mistrust in the government or the vaccine, which have been identified as major factors against cholera control [41] or vaccination in other developing countries [17], [42], did influence campaign coverage in Zanzibar only slightly. The importance of logistical issues confirms findings from a mass vaccination campaign in the cholera-endemic city of Beira, Mozambique, where main reason against OCV uptake were traveling (mentioned by 58% of non-acceptors) and being busy (26%), while the rest reported pregnancy (5.2%), refusal (3.7%), long waiting time (3.1%) and taking medication (2.6%) [8].

People's own busy daily schedules and obligations, which made it also less likely for them to receive timely information about the planned mass vaccination campaign, were limiting factors to receive vaccines. Qualitative data clearly indicate that those residents who had been away during the campaign still wished to receive the vaccine. Thus, it can be expected that an earlier start of the mobilization—media broadcasts and meetings with community leaders started only shortly before the campaign in January 2009—is likely to increase coverage because people would have more time to plan their activities around the campaign. Alternative ways to administer the vaccine may have to be considered as well to better reach those population groups that are in principal willing to get vaccinated but whose daily schedules or professional activities make it difficult to receive vaccines.

Fears about possible side effects were a substantive barrier to uptake; this needs to be addressed in future campaigns. The usually mild and transient side effects of Dukoral® (or other OCVs) [7], [38] should be explained more properly versus the benefit of protection against cholera. Such information may also re-emphasize that the vaccine is administered orally and not through injections.

Rumors about sterility have been reported in many immunization campaigns in Africa [43]. However, contrary to studies reporting that Muslims believe vaccines might cause infertility or could have been adulterated with anti-fertility agents [42]–[44], issues around fertility were not an important barrier to vaccine uptake. This suggests that future cholera campaigns in Zanzibar are somewhat less likely to suffer from such potentially sensitive issues.

The site and gender analysis of barriers to uptake showed that logistical challenges to access vaccination posts, and a tendency to question the value of vaccination against cholera, were primarily prominent among rural men. Despite differing logistical challenges, a clear demand for OCVs or a repetition of the mass vaccination campaign was reported among both genders, highlighting the local priority and demand for vaccination for cholera control in endemic areas of Zanzibar. Because sickness prevented more than one-fourth of women (regardless of site) from accessing posts or accepting the vaccine, further study may be needed to examine whether women are in general more often sick than men in Zanzibar, or whether this gender difference occurred by chance.

This study used an approach that is broadly applicable for assessing locally relevant socio-cultural features (and barriers) of vaccine acceptance in cholera-endemic areas. Because cholera control (and other disease control activities) requires consideration of local contexts, it cannot be expected that all findings are applicable locally in all other settings, even if some of them may be. It should also be borne in mind that this was a cross-sectional survey, where only associations and no causal relationships could be examined.

### Lessons learned and recommendations

Despite a high willingness to receive free vaccines, coverage was less than satisfying in the 2009 oral cholera mass vaccination campaign in Zanzibar. Complementing a pre-vaccination community survey that identified predisposing social and cultural factors as determinants of OCV uptake, this post-vaccination survey examined which social and cultural factors were associated with uptake and assessed barriers to uptake among unvaccinated community residents.

Factors associated with uptake indicated a positive impact of the mass vaccination campaign and of community sensitization activities on vaccine acceptance behavior. Unlike in other circumstances, where communities opposed cholera control or where public trust of vaccines was damaged, the evaluation of barriers to uptake also indicated a good implementation of the mass vaccination campaign and trust in the health system.

High community awareness of cholera and a positive attitude towards receiving OCVs, especially if they are provided without charge, suggest little opposition to vaccination as a supplementary means to cholera control in Zanzibar. Despite such encouraging prospects and demand for repeating vaccination in cholera-endemic populations, local decision-makers and public health officials still need to know how community coverage of mass campaigns could be improved. Even though the following recommendations are in principle limited to cholera-endemic communities in Zanzibar, national and international cholera control experts may also benefit from them, and focus groups may be useful to guide implementation of these recommendations.

First, campaigns should be announced earlier, at least a few months before vaccination posts open, with repeated reminders in the target communities. Second, campaign planners may also consider an extension of daily open hours or numbers of days for the vaccination especially in rural areas. Third, information about the campaign should not only cover dates and venues, specific requirements and inclusion criteria, but, fourth, also reinforce again more general health education on hygiene and diarrhea to interrupt fecal-oral transmission and, fifth, particularly point out the value of vaccination versus treatment of cholera with antibiotics. Sixth, although side effects of OCVs are usually mild, they should not only be specified, but also explained versus the benefit of vaccination. Finally, identification of alternative solutions to mass vaccination campaigns may be needed for population groups that recognize the value of vaccination in principal but are harder to reach due to their daily or professional activities.

## Supporting Information

Text S1 **EMIC interview for study of community views of cholera and barriers to oral cholera vaccine uptake.**
(PDF)
